# Dynamics of Blister Actuation in Laser-Induced Forward Transfer for Contactless Microchip Transfer

**DOI:** 10.3390/nano14231926

**Published:** 2024-11-29

**Authors:** DoYoung Kim, Seong Ryu, Sukang Bae, Min Wook Lee, Tae-Wook Kim, Jong-Seong Bae, Jiwon Park, Seoung-Ki Lee

**Affiliations:** 1 School of Material Science and Engineering, Pusan National University, Busan 46241, Republic of Korea; kimdo0@pusan.ac.kr (D.K.);; 2 Institute of Advanced Composite Materials, Korea Institute of Science and Technology (KIST), 92 Chudong-ro, Bongdong-eup, Wanju 55324, Republic of Korea; 3 Department of JBNU-KIST Industry-Academia Convergence Research, Jeonbuk National University, Jeonju 54896, Republic of Korea; 4 Department of Flexible and Printable Electronics, Jeonbuk National University, Jeonju 54896, Republic of Korea; 5 Busan Center, Korea Basic Science Institute, Busan 46742, Republic of Korea; 6 R & D Center of JB Lab Corporation, Seoul 08788, Republic of Korea

**Keywords:** laser-induced forward transfer, micro-light-emitting diode, blister actuation, contactless transfer, microchip

## Abstract

The rapid evolution of microelectronics and display technologies has driven the demand for advanced manufacturing techniques capable of precise, high-speed microchip transfer. As devices shrink in size and increase in complexity, scalable and contactless methods for microscale placement are essential. Laser-induced forward transfer (LIFT) has emerged as a transformative solution, offering the precision and adaptability required for next-generation applications such as micro-light-emitting diodes (μ-LEDs). This study optimizes the LIFT process for the precise transfer of silicon microchips designed to mimic μ-LEDs. Critical parameters, including laser energy density, laser pulse width, and dynamic release layer (DRL) thickness are systematically adjusted to ensure controlled blister formation, a key factor for successful material transfer. The DRL, a polyimide-based photoreactive layer, undergoes photothermal decomposition under 355 nm laser irradiation, creating localized pressure that propels microchips onto the receiver substrate in a contactless manner. Using advanced techniques such as three-dimensional profilometry, X-ray photoelectron spectroscopy, and ultrafast imaging, this study evaluates the rupture dynamics of the DRL and the velocity of microchips during transfer. Optimization of the DRL thickness to 1 µm and a transfer velocity of 20 m s⁻^1^ achieves a transfer yield of up to 97%, showcasing LIFT’s potential in μ-LED manufacturing and semiconductor production.

## 1. Introduction

The increasing demand for advanced display technologies, particularly in automotive, wearable, and augmented reality devices, has accelerated the development of flexible, rollable, and energy-efficient displays. To meet these diverse form factor requirements, micro-light-emitting diodes (μ-LEDs) are recently gaining attention as a next-generation display technology due to their superior brightness characteristics, long lifespans, and rapid response times, rendering them ideal for use in various modern applications [1,2]. However, despite their potential, the commercialization of μ-LED displays remains a challenge, primarily due to high production costs and scalability issues related to the transfer of millions of μ-LEDs onto panels. To mitigate these issues, various cost-effective transfer methods have emerged for μ-LEDs, including polydimethylsiloxane (PDMS)-based transfer, electrostatic pickup transfer, fluidic transfer, and laser-induced transfer [3,4,5]. Among these, laser-induced forward transfer (LIFT) has garnered significant attention as a promising solution to these challenges, owing to its high-speed, contamination-free, and precise transfer capabilities [6,7].

LIFT operates by using a pulsed laser to ablate a photoreactive polymer-based sacrificial layer, such as a dynamic release layer (DRL), thereby generating a gas pressure that propels materials from a donor substrate to a receiver substrate [8]. This non-contact, high-throughput transfer process is particularly well suited for applications such as μ-LED assembly, as demonstrated previously by Marinov et al. [9]. LIFT enhances transfer speeds by enabling the simultaneous transfer of multiple μ-LEDs, achieving rates exceeding 100 million units per hour, thereby addressing scalability issues and potentially lowering production costs. However, while various studies have focused on the process results of LIFT, the specific dynamic behaviors and mechanisms involved in the transfer of solid materials, particularly during the laser ablation process, remain unexplored.

In this study, we investigated the application of the LIFT process for the transfer of silicon microchips, which were designed to mimic μ-LEDs, while focusing on the intricate dynamics of blister actuation during the transfer process. For this purpose, the microchips were positioned on a transparent glass substrate coated with a photoreactive polyimide-based DRL, selected due to its exceptional photothermal properties [10]. Upon exposure to 355 nm laser irradiation, the DRL underwent photothermal decomposition, generating gaseous by-products and forming blisters that propelled the microchips to the receiver substrate in a precise contactless manner. To achieve a comprehensive understanding of the forces driving this process, three-dimensional (3D) profilometry was employed to analyze the sequential blister behavior, including rupture, under varying laser conditions. X-ray photoelectron spectroscopy (XPS) was employed to confirm the chemical transformations within the DRL, while ultrafast imaging was used to capture the time-resolved motion of the microchips during transfer, providing critical insights into their velocity and impact behavior. Finally, following meticulous optimization of the donor substrate, the transfer yield was evaluated for offering new possibilities for LIFT application in μ-LED display fabrication and semiconductor technologies.

## 2. Materials and Methods

### 2.1. Materials

Polyamic acid solution (80% NMP/20% aromatic hydrocarbon), hydrofluoric acid (ACS reagent, 48%) were purchased from Sigma-Aldrich (St. Louis, MO, USA) and used without further purification. Carrier glass (ultraviolet transmittance 95%) with size of 2 mm × 2 mm and thickness of 0.1 mm, 6 inch SOI wafer with top layer thickness of 5 µm and oxide layer thickness of 1 µm were purchased. For the transfer process, thermal release tape and PDMS (Sylgard 184, Dow Corning, Midland, MI, USA) was purchased.

### 2.2. Donor Substrate Preparation

Polyamic acid solution was spin-coated on the carrier glass using a two-step process: 1000 rpm for 20 s followed by 3000–7000 rpm for 120 s to modify the thickness. The thickness of PI was measured using an alpha-step. After spin-coating, it was partially cured on a hot-plate at 100 °C for 5–80 min to control the embedding depth of microchips within the polyimide. Microchip arrays were prepared by creating a hole pattern by the usage of photolithography. After patterning holes on the top layer of the SOI wafer, Inductively Coupled Plasma Reactive Ion Etching (ICP-RIE) was performed to etch the top silicon layer using the Bosch process (ICP 400 W, Platen 5 W, SF_6_, O_2_, C_4_F_8_ gas atmosphere) and to expose the underlying oxide layer [11]. Subsequently, the SiO_2_ layer was removed by wet etching using HF [12]. Thermal release tape was used to pick up the top layer, which was then transferred onto a pre-cured polyimide-glass substrate [13]. During this process, microchips sinks into the polyimide which ensures well embedding from 0.1 μm to 0.9 μm. The polyimide had been partially cured to ensure good adhesion, and the release of the top layer was completed by applying uniform pressure on a 110 °C hot-plate for 30 s. A weight of 100 g was placed on top to ensure even contact and successful transfer of the layer followed by the full curing process of polyimide on a vacuum for 2 h on 200 °C. Finally, microchip array was patterned with photolithography and etched with ICP-RIE.

### 2.3. Receiver Substrate Preparation

PDMS was prepared as the receiver substrate by mixing the prepolymer and curing agent in a 10:1 ratio [14]. The elastic modulus of PDMS was primarily controlled by the ratio of prepolymer to curing agent, with a higher prepolymer content resulting in a softer and more elastic material [15]. This elasticity not only enhances the adhesive capability of the PDMS for capturing microchips but also helps absorb impact forces during the transfer process. 

### 2.4. Laser-Induced Forward Transfer

The donor and receiver substrates were positioned facing each other on a specially designed optical alignment stage, capable of movement in the x, y, and z axes, as well as rotation. A pulsed UV laser (λ = 355 nm) was irradiated to the interface between the DRL and the carrier glass to transfer microchip arrays into the receiver substrate. The laser energy density was controlled through modulation of the laser’s frequency (130–250 kHz), pulse width (1–3.8 µs), and focus depth (0.1 mm–0.3 mm). Continuous measurements of the laser energy density were performed using a Thorlab thermal power detector, with the laser beam being consistently irradiated during measurement. 

### 2.5. Morphology Analysis

3D images of the blisters and step height were characterized using a 3D optical profilometer. The Profilm3D optical profiler utilized white light interferometry (WLI) for non-contact surface measurements. This method provided precise surface profiles and ensured detailed characterization of the blisters and step height. Cross-sectional image of was obtained by Cryo-Focused Ion Beam-Scanning Electron Microscope. 

### 2.6. X-Ray Photoelectron Spectroscopy (XPS) Analysis

The chemical state study of etch elements were performed High-Performance X-ray Photoelectron Spectroscopy: HP-XPS (BS101, K-ALPHA^+^, Thermo Fisher Scientific Inc. (Oxford, UK)) using monochromated Al Kα X-ray source (hν = 1486.6 eV, power = 12 kV, 72 W) at a spot size of 400 µm in diameter with charge compensation using two flood gun (low energy electron and Ar+ ion) at the Busan Center of Korea Basic Science Institute (KBSI).

## 3. Results and Discussion

**Figure 1:**
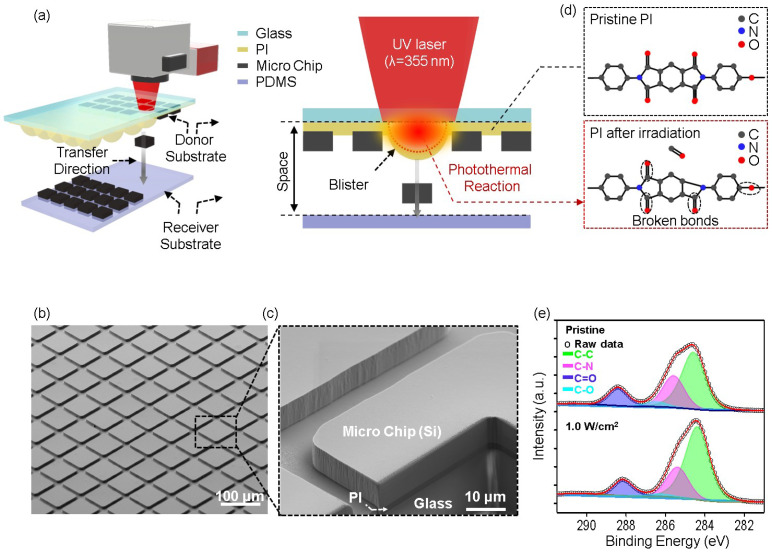
(**a**) Schematic of the LIFT process, (**b**) SEM image of the microchip array, (**c**) FIB cross-sectional image of the microchip, (**d**) schematic illustration of the photothermal reaction at the PI–glass interface, showing breakage of the C=O and C–O bonds and subsequent gas generation, and (**e**) XPS data recorded for the PI before and after laser irradiation.

The full LIFT process is schematically shown in Figure 1a. This process requires a donor substrate, which consists of microchips attached to an ultraviolet (UV)-reactive photopolymer layer known as the DRL. The DRL plays a crucial role in facilitating the separation or transfer of material upon laser irradiation. For the purpose of this study, polyimide (PI) was selected as the DRL material due to its strong photoreactive properties, which render it highly suitable for achieving clean and efficient ablation under UV laser exposure [16,17,18]. The PI layer was coated onto a transparent carrier glass substrate. Figure 1b displays the scanning electron microscopy (SEM) image of the donor substrate, showing an array of microchips partially embedded in the DRL. Each microchip measures 50 μm × 50 μm, with a spacing of 5 μm between adjacent chips. 

To further investigate the structures and embedded depths of the microchips, a focused ion beam (FIB) cross-section image was captured (Figure 1c), revealing that a shallow portion of the microchip was securely embedded in the DRL. This embedded depth determines the adhesion strength with which the microchip is attached to the donor layer.

Opposite the donor substrate, a receiver substrate with a silicone-based adhesive surface, i.e., PDMS, was positioned to capture the microchips during the transfer process. This adhesive layer ensures secure attachment of the microchips following transfer. Subsequently, the LIFT process was initiated by directing a pulsed UV laser with a wavelength of 355 nm through the carrier glass, which possesses a transmittance of 95% at this wavelength. The laser beam specifically targets the interface between the carrier glass and the DRL, causing rapid localized heating [19]. This heat induces a photothermal reaction, leading to the breakage of chemical bonds within the PI, particularly C=O and C–O bonds [20]. As these bonds break, gaseous by-products such as carbon monoxide (CO) are generated at the interface between the PI and the carrier glass, as shown in Figure 1d [10,21,22]. As the gas accumulates at the interface between the PI and the glass layer, pressure builds up, causing the PI layer to expand and form blisters. The rapid expansion of these blisters provides the force necessary to propel the microchips away from the DRL surface and onto the receiver substrate in a precise, non-contact manner. During this process, it is essential that the laser energy is carefully controlled, as excessive pressure within the DRL can lead to rupture of the blisters, resulting in contamination of the transferred chips.

To further investigate the chemical changes occurring in the DRL during laser irradiation, X-ray photoelectron spectroscopy (XPS) was employed, and the results are presented in Figure 1e. The high-resolution C 1s spectra were analyzed to evaluate the bonding environments within the PI both before and after laser treatment. Curve fitting of the C 1s peak was carried out using CasaXPS 2.3.26 software, and all spectra were referenced to the C 1s signal at 284.6 eV (corresponding to adventitious carbon) to ensure accurate comparison. The XPS spectra reveal distinct changes in the chemical composition of the PI following laser irradiation. In the pristine PI, deconvolution of the C 1 s peak shows contributions from several functional groups, including C–C bonds at 284.55 eV, C–N bonds at 285.5 eV, C–O bonds at 286.45 eV, and C=O bonds within the imide ring at 288.31 eV. Upon exposure to the UV laser at an energy density of 1 W cm^−2^, significant reductions in the C–N, C–O, and C=O peak intensities compare to C–C bonds were observed, indicating the breakdown of these bonds during the photothermal ablation process [23]. More specifically, the reduction in the C–N and C=O peak intensities suggests that laser irradiation induces decomposition of the PI structure, viz., cleavage of the carbonyl (C=O) and ether (C–O) bonds. This decomposition results in the release of volatile species, such as CO and carbon dioxide (CO_2_), which are responsible for gas generation at the PI–glass interface [24]. These gaseous products contribute to the pressure build up within the DRL, which ultimately drives blister formation and facilitates transfer of the microchips. The XPS data corroborate the proposed mechanism of laser-induced decomposition and gas evolution, further highlighting the critical role of chemical bond cleavage in the dynamic release process. The observed chemical modifications in the DRL are directly linked to the efficient material transfer that occurs during the LIFT process. This blister formation is crucial for transferring material from the donor substrate to the receiver substrate during the LIFT process.

**Figure 2:**
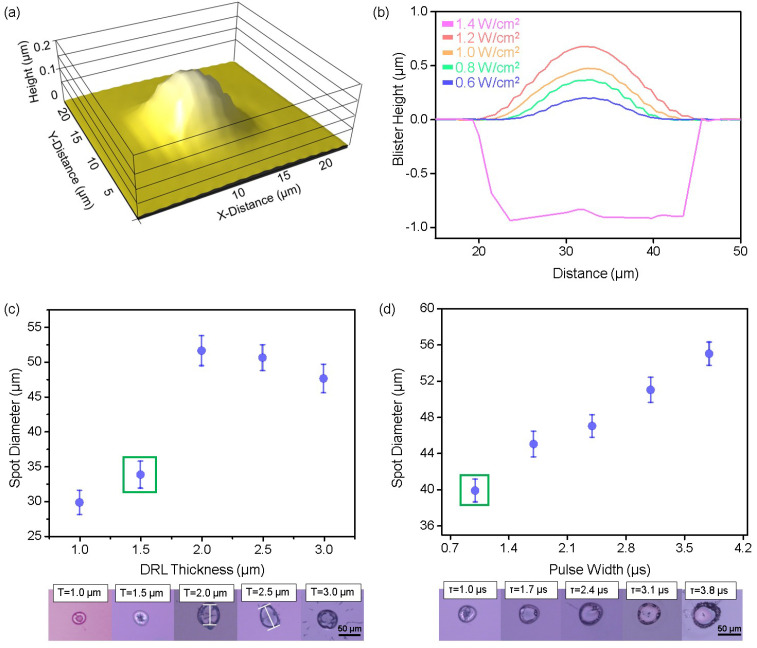
(**a**) 3D Profiler image of a blister and (**b**) comparison of blister heights (red, orange, green, and blue) and rupture sizes (pink). The spot diameter is also given as a function of (**c**) the DRL thickness and (**d**) the laser pulse width. The error bars represent the standard deviations.

Figure 2a shows the representative 3D structure of a blister, which consists of a hemispherical bulge with the highest point at its center. The blister formed at a laser energy density of 0.6 W cm^−2^, which is above the blistering threshold, was found to possess a diameter of 15 μm and a height of 0.2 μm [25,26,27]. This result indicates the ability of the laser to generate stable blisters under controlled conditions. As demonstrated in Figure 2b, the blister formation behavior depends strongly on the laser energy density [28]. Upon increasing the laser energy density from 0.6 to 1.2 W cm^−2^, the height of the blister in the DRL (thickness, t = 1 μm) increased accordingly. This result suggests a direct relationship between laser energy density and blister formation, as higher energy densities lead to greater expansion within the DRL [29,30]. However, when the energy density exceeded 1.4 W cm^−2^, the excessive energy input resulted in rupture of the DRL, as shown in Figure S2 [31]. This finding underscores the importance of identifying an appropriate laser energy density range to promote blister formation without damaging the donor layer because an overly high energy input can compromise the structural integrity of the DRL [19]. In addition, since the residue dispersed by rupture can contaminate the microchip to be transferred, it is important to operate above the blistering limit while remaining below the rupture limit to transfer the microchip without such contamination [32]. To further explore the parameters affecting blistering, the laser energy density was fixed at 1 W cm^−2^, and the DRL thickness (t) and laser pulse width (τ) were adjusted, as shown in Figure 2c,d. Figure 2c shows the relationship between the DRL thickness and the dimensions of laser-responsive region (i.e., blister or rupture). It can be seen that the dimensional behavior of the laser-responsive region varied significantly with an increasing DRL thickness under fixed laser conditions. More specifically, when the DRL thickness reached 2 μm, the diameter of the laser-affected area increased. However, at thicknesses beyond 2 μm, rupture occurred, causing the diameter to decrease. Blister formation was observed only at a DRL thickness of 1.5 μm (marked in green), with both thicker and thinner layers leading to rupture. This can be explained by the thermal and mechanical properties of the DRL. For example, at 1.5 μm, the laser energy is efficiently absorbed and converted into localized expansion, generating sufficient pressure to induce blister formation without exceeding the mechanical limits of the DRL. This layer is sufficiently thick to avoid rupture but forms localized heat accumulation. For DRL thicknesses >2 μm, the absorbed energy is distributed across a larger volume, leading to an increase in the internal pressure, and eventual rupture as the mechanical strength of the material is exceeded. This rupture event reduces the effective blister size as the structure collapses. Conversely, when the DRL is thinner than 1.5 μm, the layer lacks the mechanical stability to withstand localized pressure, resulting in rupture despite an adequate energy input. Furthermore, this thin layer concentrates heat, leading to intense stress and failure before a blister can fully develop [19,33]. These findings highlight the importance of the DRL thickness in ensuring stable blister formation. Indeed, optimization of the DRL thickness ensures efficient energy absorption and expansion without rupture, facilitating reliable material transfer during the LIFT process.

As another laser parameter, the effect of the laser pulse width (τ) on the blister/rupture behavior was examined at a DRL thickness of 1.5 μm. As shown in Figure 2d, blister formation occurs at a pulse width of 1 μs (marked in green). The energy deposition over this short timeframe effectively concentrates the thermal energy, creating sufficient pressure to propel the material (e.g., the microchips) onto the receiver substrate in a controlled manner [34]. As the pulse width increases beyond 1 μs, the size of the laser-responsive region expands due to the longer interaction time between the laser and the PI. This leads to increased heat diffusion and a larger volume of DRL being affected by the laser. Consequently, the thermal energy spreads over a wider area within the DRL, reducing the rate of localized pressure build up, but still allowing excess pressure to accumulate over time. The extended interaction time promotes more significant internal gas generation, which leads to a sudden and uncontrolled release of gas, resulting in rupture rather than controlled blister formation [35]. At longer pulse widths (τ > 1 μs), the slower rate of energy deposition enables the heat to penetrate deeper into the DRL, further affecting its mechanical stability [36]. This prolonged exposure can cause material degradation within the DRL, weakening its structural integrity and rendering it more prone to rupture under excessive internal pressure. Table S1 provides comparative analysis on the parameters affecting blister formation in this study. These findings suggest that the pulse width directly affects both the thermal dynamics and mechanical stability of the DRL, indicating its critical role during optimization of the LIFT process.

**Figure 3:**
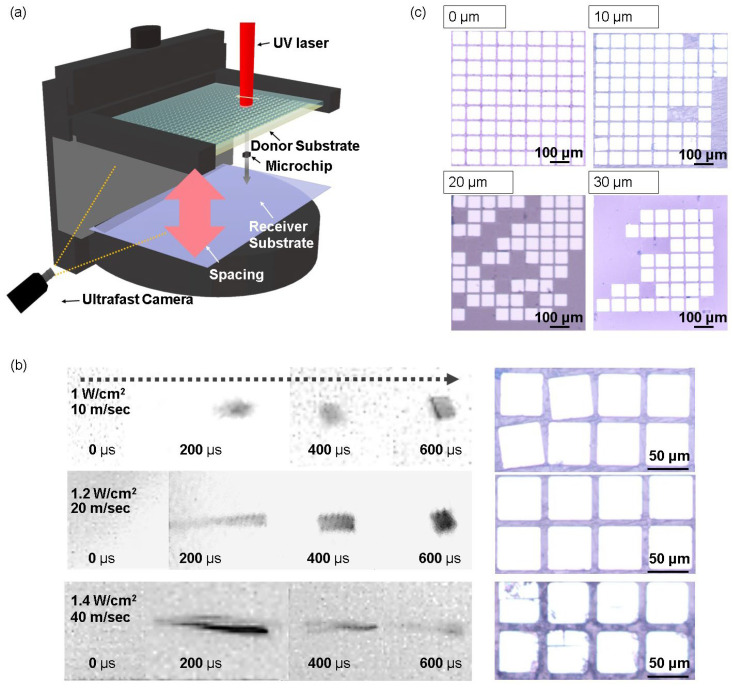
(**a**) Schematic representation of the custom-designed optical alignment stage for the non-contact LIFT process. (**b**) Series of time-resolved images at different laser energy densities. The corresponding OM images show the transferred microchips on the receiver substrates. (**c**) OM images of the receiver substrate with varying spacings.

Considering the influence of different laser parameters on the structural behavior of blistering in the DRL (Figure 2), a non-contact LIFT process was implemented for microchips. A custom-made optical alignment stage was engineered to facilitate this process, providing precise adjustments along the x, y, and z axes, as well as rotational control (Figure 3a and Figure S3). The upper section of the stage (Part A) secures the donor substrate, allowing for fine positional adjustments to ensure accurate alignment during transfer. The lower section (Part B) accommodates the receiver substrate and offers rotational capabilities to further enhance alignment precision. This stage enables meticulous tuning of the substrate position, ensuring stable, repeatable, and accurate transfer outcomes throughout the LIFT process. The three sets of time-resolved images shown in Figure 3b represent the motion of the microchip at three distinct average flying velocities (i.e., 10, 20, and 40 m s^−1^) by adjusting laser energy density (1~1.4 W cm^−2^)**,** allowing a comparison of transfer behaviors to be performed under these conditions [25,37,38]. At a laser density of 1 W cm^−2^, the microchip exhibited rapid acceleration within the first 100 µs of flight, reaching its peak velocity before beginning to decelerate significantly after 200 µs, indicating a clear loss of kinetic energy. When the laser density was increased to 1.2 W cm^−2^, the microchip maintained its speed for a longer duration (0–400 µs) before undergoing a more gradual deceleration between 400 and 600 µs. In contrast, at 1.4 W cm^−2^**,** the microchip sustained a high acceleration and velocity throughout the observed time period, even at the 600 µs mark. This controlled speed suggests a more balanced dissipation of energy, which could potentially reduce the risk of damage during transfer to the receiver substrate. Optical microscopy (OM) and time-resolved imaging were then employed to depict microchip transfer to the receiver substrate with a 10 µm spacing between the donor and receiver substrates. For transfer yield analysis, we used Python 3.11 and ImageJ 1.54k software, utilizing its image sensing function to analyze whether the microchips were misaligned after transfer (as shown in Figure S4). To facilitate this analysis, a grid was overlaid on the array as a reference framework, enabling precise measurement of deviations in distance and angle. The analysis revealed that while the initial spacing between chips on the donor substrate was 8 µm, the misaligned chips exhibited a deviation of 2 µm in spacing. Additionally, angular misalignment was evaluated, and chips that rotated beyond 5° threshold were categorized as misaligned [9]. At a laser density of 1 W cm^−2^, the microchips were transferred without damage, but misalignment occurred, likely due to air resistance affecting the microchips during flight. In contrast, the OM image of the transfer conducted at 1.2 W cm^−2^ shows successful, damage-free, and well-aligned transfers. However, at 1.4 W cm^−2^, the lack of sufficient deceleration before impact caused the microchips to reach the substrate at a high velocity, resulting in potential damage or failure upon impact. These observations emphasize the critical role of the transfer velocity in achieving optimal results [39]. Thus, at energy density, such as 1 W cm^−2^, damage may be avoided, but misalignment can occur due to the influence of air resistance. Conversely, at higher energy, i.e., 1.4 W cm^−2^, the microchip cannot decelerate adequately, increasing the risk of damage during transfer. In contrast, 1.2 W cm^−2^ of energy, although relatively high, allows the microchip to overcome air resistance, maintain alignment, and reduce the impact force, leading to successful, damage-free transfer.

Furthermore, Figure 3c demonstrates how the transfer quality (e.g., alignment and yield) can be affected by the spacing between the donor and receiver substrates. For the purpose of this study, the spacing was controlled, varying from direct contact to 30 µm. When the substrates were in direct contact with one another (i.e., contact mode), the LIFT process achieved a high transfer yield. However, it has previously been reported that ensuring a small but controlled spacing between the donor and receiver substrates is essential for consistency in scalable LIFT applications, particularly for industrial applications [40]. Thus, while contact-based transfers may initially provide higher yields, they pose additional risks, such as substrate damage, contamination, and an inconsistent transfer quality, especially during mass production. By introducing appropriate spacing (i.e., non-contact mode), these issues can be mitigated, resulting in more consistent and repeatable transfers. 

However, as the spacing was increased from 10 to 30 µm, the transfer yield decreased to 42.7%. This reduction in yield can be attributed to multiple factors, including air resistance and the rotational momentum of the microchips [41,42]. When the laser is not perfectly centered on the microchip, rotational motion occurs, and as the gap widens, the rotational angle increases due to the longer flight time [43]. This enhanced rotation often leads to misalignment upon landing, further diminishing the transfer yield.

**Figure 4:**
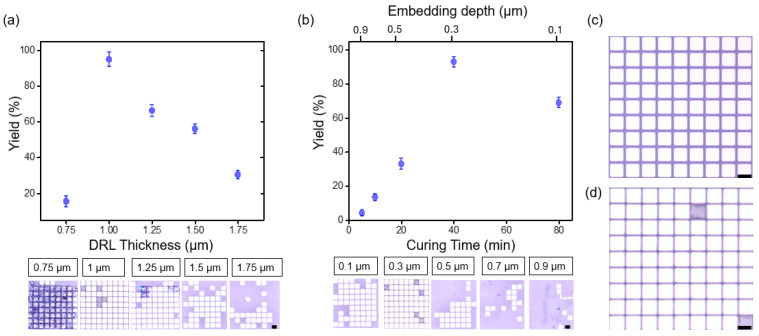
Transfer yield as a function of (**a**) the DRL thickness and (**b**) the curing time, with the corresponding embedding depth indicated on the secondary X-axis above. The error bars in these graphs represent the standard deviations. OM images of (**c**) the donor substrate before transfer and (**d**) the receiver substrate after transfer with a 97% transfer yield. The black scale bar corresponds to 100 μm.

While narrowing the spacer gap can improve the transfer yield, the aim of this study was to optimize the physical properties and structural configuration of the donor substrate to further enhance the results. Thus, Figure 4 presents the results of experiments performed to impart additional precise control on the donor substrate and improve the LIFT process, specifically in the case where the microchips are embedded within the DRL. Two key parameters of the donor substrate were optimized to achieve higher transfer yields, namely the DRL thickness and the embedding depth of the microchips within the DRL. The LIFT process was conducted on microchip arrays at a laser energy density of 1.2 W cm^−2^ with 23.5 μJ of pulse energy. Laser irradiation on the microchips utilized a scanning method with single exposure, achieved through a focusing lens that produced a spot size of 50 µm. A detailed analysis of transfer yield and classification of microchips into categories (successful transfers, misaligned transfers, and failed transfers) is illustrated in Figure S4. To provide further clarity regarding the experimental setup, the laser scanning speed was 500 mm/s, corresponding to a calculated chip-to-chip scan rate of approximately 16 μs. This setup highlights the precision and efficiency of the scanning method employed during the LIFT process. Additionally, the focused beam ensured uniform energy distribution across the microchip arrays, minimizing defects caused by uneven irradiation. For a visual depiction of the experimental configuration, including the focused beam and alignment mechanism, Figure S1 in the Supplementary Materials serves as a reference. In support of these observations, Figure S5a presents additional analyses, where transfer yield was plotted as a function of the embedding depth of the microchip within the DRL, as well as the embedding depth-to-DRL thickness ratio. These results provide deeper insights into how variations in the embedding depth of the microchip and DRL thickness influence transfer efficiency under different laser energy density conditions. Furthermore, Figure S5b complements this analysis with plots showing the correlation between DRL thickness and transfer yield under varying laser energy densities. The results reveal that each DRL thickness has an optimal laser energy density, beyond which the transfer mechanism transitions from blister-mediated transfer to rupture-induced transfer, resulting in a gradual decline in yield. For thinner DRL layers (e.g., 0.75 µm), transfer yield increases as the energy density approaches the optimal value. However, maintaining high transfer yields at higher energy densities requires thicker DRL layers. These findings underscore the importance of identifying the optimal laser energy density for each DRL thickness to maximize and sustain high transfer yields. This highlights the need for fine-tuning laser parameters to ensure broader applicability across diverse DRL configurations and material conditions. As shown in Figure 4a, optimization of the DRL thickness had a significantly impact on the microchip transfer yield. More specifically, in cases where the DRL was too thick (t > 1.5 µm), excess energy absorption led to considerable pressure build up, which resulted in excessive energy being transferred to the microchip. Eventually, the microchip was unable to settle stably on the receiver substrate and bounced off, reducing the transfer efficiency. Conversely, when the DRL was too thin (t < 0.75 µm), premature rupture, as discussed in Figure 2c, not only disrupted blister formation but also resulted in contamination of the transferred microchips, thereby reducing the overall yield. Notably, an optimal DRL thickness of 1 µm produced the highest transfer yield, reaching up to 97%. The embedding depth of the microchips within the DRL, which influences the adhesion properties between the microchips and the DRL, was further optimized by controlling the pre-curing time of the DRL precursor, as shown in Figure 4b. In this experiment, the DRL thickness was fixed at the optimized value of 1 µm. During the early stages of curing, the PI remains in a semi-fluid state, allowing the microchips to embed more deeply within the DRL. As the curing process progresses and the PI solidifies, the increased stiffness restricts further embedding, resulting in shallower placements, with microchips embedding at depths of up to 0.9 µm [44,45]. This variation in the embedding depth directly was found to affect the blistering dynamics and the transfer efficiency. To further illustrate this relationship, Table S3 has been included, providing a comparative analysis of the effect of DRL curing time on the embed depth of microchips. Moreover, the results presented in Figure 4b show that shorter curing times (5–10 min) result in deeper microchip embedding, which impairs efficient release during the LIFT process and lowers the transfer yield. As the curing time was increased, the embedding depth decreased up to 0.1 µm, positioning the microchips more favorably for release. However, excessive curing times led to insufficient embedding, weakening adhesion between the microchips and the DRL, and resulting in microchip loss prior to transfer [46]. The highest transfer yield was achieved at a curing time of ~40 min, with embedding depth of 0.3 µm at which point the embedding depth provided an optimal balance for effective release. Table S2 summarizes a comparative evaluation of the parameters influencing transfer yield in this study. Figure 4c presents an OM image of the donor substrate bearing an array of microchips, while Figure 4d shows the corresponding image for the receiver substrate with a transfer yield of ~97%. These images confirm the successful, defect-free transfer of microchips, made possible by precise optimization of the DRL thickness and the microchip embedding depth. Overall, this study demonstrates the critical influence of the thickness and curing time on controlling the microchip embedding depth, which maximizes the transfer efficiency by preventing issues related to inadequate or excessive embedding.

## 4. Conclusions

This study presents a comprehensive exploration of the laser-induced forward transfer (LIFT) process, with a focus on the optimizing laser parameters and the donor substrate characteristics to achieve more efficient and reliable microchip transfer. By fine-tuning key variables, such as the laser energy density, the dynamic release layer (DRL) thickness, and the pulse width, precise control was achieved over blister formation, which is essential for avoiding contamination and blister rupture. Notably, a DRL thickness of 1 µm provided the ideal balance for achieving stable blister dynamics. Ultrafast imaging revealed that a transfer velocity of 20 m s^−2^ offered the optimal trade-off between the air resistance and the impact force, ensuring accurate and damage-free microchip placement. Consequently, the optimized process achieved a maximum transfer yield of ~97%, representing a substantial improvement in efficiency compared to previous systems. In practical applications of micro LED transfer, a post-transfer soldering process is typically necessary to establish reliable electrical connections between the micro LEDs and the receiver substrate [9]. With further development of post-process for practical micro LED applications, the LIFT process could be fully realized as a complete method for micro LED fabrication. These findings offer critical insights for advancing the LIFT process, thereby rendering it a promising technique for scalable, high-precision applications in micro-light-emitting diode displays and semiconductor devices.

## Data Availability

Data are contained within this article and Supplementary Materials.

## Supplementary Materials

The following supporting information can be downloaded at: https://www.mdpi.com/article/10.3390/nano14231926/s1, Figure S1: Schematic of LIFT process with substrate preparation; Figure S2: 3D profiler image of ruptured donor substrate on laser irradiated spot; Figure S3: A custom-built optical alignment stage designed to facilitate precise control during the LIFT process; Figure S4: Classification and analysis of transferred microchips; Figure S5: Analysis on key parameters influencing transfer yield; Table S1: Comparison of parameters for blister formation; Table S2: Comparison of parameters for transfer yield; Table S3: Comparative analysis of the effect of DRL curing time on the embed depth of microchips.

## References

[1] Ryu J.E., Park S., Park Y., Ryu S.W., Hwang K., Jang H.W. (2023). Technological breakthroughs in chip fabrication, transfer, and color conversion for high-performance micro-LED displays. Adv. Mater..

[2] Anwar A.R., Sajjad M.T., Johar M.A., Hernández-Gutiérrez C.A., Usman M., Łepkowski S. (2022). Recent progress in micro-LED-based display technologies. Laser Photonics Rev..

[3] Chen F., Bian J., Hu J., Sun N., Yang B., Ling H., Yu H., Wang K., Gai M., Ma Y. (2022). Mass transfer techniques for large-scale and high-density microLED arrays. Int. J. Extrem. Manuf..

[4] Lee D., Cho S., Park C., Park K.R., Lee J., Nam J., Ahn K., Park C., Jeon K., Yuh H. (2023). Fluidic self-assembly for MicroLED displays by controlled viscosity. Nature.

[5] Lu H., Guo W., Su C., Li X., Lu Y., Chen Z., Zhu L. (2020). Optimization on adhesive stamp Mass-transfer of micro-LEDs with support vector machine model. IEEE J. Electron. Devices Soc..

[6] Serra P., Piqué A. (2019). Laser-induced forward transfer: Fundamentals and applications. Adv. Mater. Technol..

[7] Goodfriend N.T., Heng S.Y., Nerushev O.A., Gromov A.V., Bulgakov A.V., Okada M., Xu W., Kitaura R., Warner J., Shinohara H. (2018). Blister-based-laser-induced-forward-transfer: A non-contact, dry laser-based transfer method for nanomaterials. Nanotechnology.

[8] Moreno-Labella J., Munoz-Martin D., Marquez A., Morales M., Molpeceres C. (2020). Simulation of direct and blister-assisted laser-induced forward transfer techniques. Procedia CIRP.

[9] Marinov V.R. 52-4: Laser-Enabled Extremely-High Rate Technology for µLED Assembly. Proceedings of the SID Symposium Digest of Technical Papers.

[10] Lan K., Deng Y., Huang A., Li S.-Q., Liu G., Xie H.-L. (2022). Highly-performance polyimide as an efficient photothermal material for solar-driven water evaporation. Polymer.

[11] Chen S.-C., Lin Y.-C., Wu J.-C., Horng L., Cheng C. (2007). Parameter optimization for an ICP deep silicon etching system. Microsyst. Technol..

[12] Park J.K., Zhang Y., Xu B., Kim S. (2021). Pattern transfer of large-scale thin membranes with controllable self-delamination interface for integrated functional systems. Nat. Commun..

[13] Bae S., Kim H., Lee Y., Xu X., Park J.-S., Zheng Y., Balakrishnan J., Lei T., Ri Kim H., Song Y.I. (2010). Roll-to-roll production of 30-inch graphene films for transparent electrodes. Nat. Nanotechnol..

[14] Kim M., Moon B.-U., Hidrovo C.H. (2013). Enhancement of the thermo-mechanical properties of PDMS molds for the hot embossing of PMMA microfluidic devices. J. Micromech. Microeng..

[15] Schneider F., Draheim J., Kamberger R., Wallrabe U. (2009). Process and material properties of polydimethylsiloxane (PDMS) for Optical MEMS. Sens. Actuators A.

[16] Papavlu A.P., Lippert T. (2018). LIFT Using a Dynamic Release Layer. Laser Printing of Functional Materials.

[17] Lippert T. (2009). UV laser ablation of polymers: From structuring to thin film deposition. Laser-Surface Interactions for New Materials Production: Tailoring Structure and Properties.

[18] Lippert T., David C., Dickinson J., Hauer M., Kogelschatz U., Langford S., Nuyken O., Phipps C., Robert J., Wokaun A. (2001). Structure property relations of photoreactive polymers designed for laser ablation. J. Photochem. Photobiol. A.

[19] Hecht L., Rager K., Davidonis M., Weber P., Gauglitz G., Dietzel A. (2019). Blister-actuated LIFT printing for multiparametric functionalization of paper-like biosensors. Micromachines.

[20] Dong Z., He Q., Shen D., Gong Z., Zhang D., Zhang W., Ono T., Jiang Y. (2023). Microfabrication of functional polyimide films and microstructures for flexible MEMS applications. Microsyst. Nanoeng..

[21] Ortelli E., Geiger F., Lippert T., Wei J., Wokaun A. (2000). UV-laser-induced decomposition of Kapton studied by infrared spectroscopy. Macromolecules.

[22] Jing P., Zhou X., Xu Z., Xu Z. (2022). Numerical and experimental investigation on photothermal performance of polyimide/high-electrical-performance-coating composite films considering surface roughness. J. Therm. Sci..

[23] Bian J., Chen F., Ling H., Sun N., Hu J., Huang Y. (2022). Experimental and modeling study of controllable laser lift-off via low-fluence multiscanning of polyimide-substrate interface. Int. J. Heat Mass Transf..

[24] Yung K.C., Zeng D., Yue T.M. (2001). XPS investigation of Upilex-S polyimide ablated by 355 nm Nd: YAG laser irradiation. Appl. Surf. Sci..

[25] Yusupov V., Churbanov S., Churbanova E., Bardakova K., Antoshin A., Evlashin S., Timashev P., Minaev N. (2020). Laser-induced forward transfer hydrogel printing: A defined route for highly controlled process. Int. J. Bioprinting.

[26] Bityurin N., Luk’Yanchuk B., Hong M., Chong T. (2003). Models for laser ablation of polymers. Chem. Rev..

[27] Metayer P., Davenas J., Bureau J. (2001). Ablation and carbon deposition induced by UV laser irradiation of polyimide: Application to the metallization of VIAs in high density printed circuit boards. Nucl. Instrum. Methods Phys. Res. Sect. B.

[28] Chang J., Sun X. (2023). Laser-induced forward transfer based laser bioprinting in biomedical applications. Front. Bioeng. Biotechnol..

[29] Godfrey A.T., Kallepalli D.L., Ratté J., Zhang C., Corkum P. (2020). Femtosecond-laser-induced nanoscale blisters in polyimide thin films through nonlinear absorption. Phys. Rev. Appl..

[30] Hong J. (2019). Thermo-mechanical analysis of blister formation on a rigid substrate in blister-actuated laser-induced forward transfer. IEEE Trans. Compon. Packag. Manuf. Technol..

[31] Zhang T., Wu C., Rong Y., Li M., Huang Y., Zhang G. (2022). Laser ablation behavior and mechanism of polyimide by UV irradiation. Mater. Manuf. Process..

[32] Brown M.S., Kattamis N.T., Arnold C.B. (2010). Time-resolved study of polyimide absorption layers for blister-actuated laser-induced forward transfer. J. Appl. Phys..

[33] Fardel R., Nagel M., Nüesch F., Lippert T., Wokaun A. (2007). Laser forward transfer using a sacrificial layer: Influence of the material properties. Appl. Surf. Sci..

[34] Marinov V.R., Swenson O., Atanasov Y., Schneck N. (2013). Laser-assisted ultrathin die packaging: Insights from a process study. Microelectron. Eng..

[35] Kattamis N.T., Brown M.S., Arnold C.B. (2011). Finite element analysis of blister formation in laser-induced forward transfer. J. Mater. Res..

[36] Bornemann S., Yulianto N., Spende H., Herbani Y., Prades J.D., Wasisto H.S., Waag A. (2020). Femtosecond Laser Lift-Off with Sub-Bandgap Excitation for Production of Free-Standing GaN Light-Emitting Diode Chips. Adv. Eng. Mater..

[37] Li Q., Grojo D., Alloncle A.-P., Delaporte P. (2019). Jetting regimes of double-pulse laser-induced forward transfer. Opt. Mater. Express.

[38] Hu Y., Cheng H., Xu J., Yao Z. (2017). A coupling model to simulate the dynamic process of blister-actuated nanosecond laser-induced forward transfer. J. Phys. D.

[39] Shaw-Stewart J., Lippert T., Nagel M., Nüesch F., Wokaun A. (2012). A simple model for flyer velocity from laser-induced forward transfer with a dynamic release layer. Appl. Surf. Sci..

[40] Smits E.C., Walter A., De Leeuw D.M., Asadi K. (2017). Laser induced forward transfer of graphene. Appl. Phys. Lett..

[41] Pohl R., Jansink M., Römer G., Huis in ‘t Veld A. (2015). Solid-phase laser-induced forward transfer of variable shapes using a liquid-crystal spatial light modulator. Appl. Phys. A.

[42] Araki T., den Toonder J.M., Suganuma K., Uemura T., Noda Y., Yoshimoto S., Izumi S., Sekitani T. (2019). Non-contact laser printing of ag nanowire-based electrode with photodegradable polymers. J. Photopolym. Sci. Technol..

[43] Vogel A., Lorenz K., Horneffer V., Hüttmann G., Von Smolinski D., Gebert A. (2007). Mechanisms of laser-induced dissection and transport of histologic specimens. Biophys. J..

[44] Daissè G., Marcon M., Zecchini M., Wan-Wendner R. (2022). Cure-dependent loading rate effects on strength and stiffness of particle-reinforced thermoset polymers. Polymer.

[45] Weidmann G., Ogorkiewicz R. (1974). Effects of time, temperature and curing on the stiffness of epoxy laminating systems. J. Mater. Sci..

[46] Sano T., Yamada H., Nakayama T., Miyamoto I. (2002). Experimental investigation of laser induced forward transfer process of metal thin films. Appl. Surf. Sci..

