# Supplementary material for: Dynamics of Blister Actuation in Laser-Induced Forward Transfer for Contactless Microchip Transfer
Source: Nanomaterials (Basel). 2024 Nov 29;14(23):1926. doi: 10.3390/nano14231926 (PMC11643012; doi:10.3390/nano14231926)
Supplement: Supplementary file 1 [file nanomaterials-14-01926-s001.zip › nanomaterials-3303283-supplementary.pdf]

Supplementary Materials

Dynamics of Blister Actuation in Laser-Induced Forward Transfer for Contactless Micro-Chip transfer

DoYoung Kim[1], Seong Ryu[1], Sukang Bae[2,3], Min Wook Lee[2], Tae-Wook Kim[3,4], Jiwon Park[5], Jong-Seong Bae[6,*], and Seoung-Ki Lee[1, *]

[1]School of Material Science and Engineering, Pusan National University, Busan 46241, Republic of Korea

[2]Institute of Advanced Composite Materials, Korea Institute of Science and Technology (KIST), 92 Chudong-ro, Bongdong-eup, Wanju-gun, Jeonbuk-do, 55324, Republic of Korea

[3] Department of JBNU-KIST Industry-Academia Convergence Research, Jeonbuk National University, Jeonju-si 54896, Republic of Korea

[4] Department of Flexible and Printable Electronics, Jeonbuk National University, Jeonju-si 54896, Republic of Korea

[5]R & D Center of JB Lab Corporation, Seoul 08788, Republic of Korea

[6]Busan Center, Korea Basic Science Institute, Busan 46742, Republic of Korea

*Corresponding Author

E-mail: jsbae@kbsi.re.kr, ifriend@pusan.ac.kr

Figure S1. Schematic of LIFT process with substrate preparation

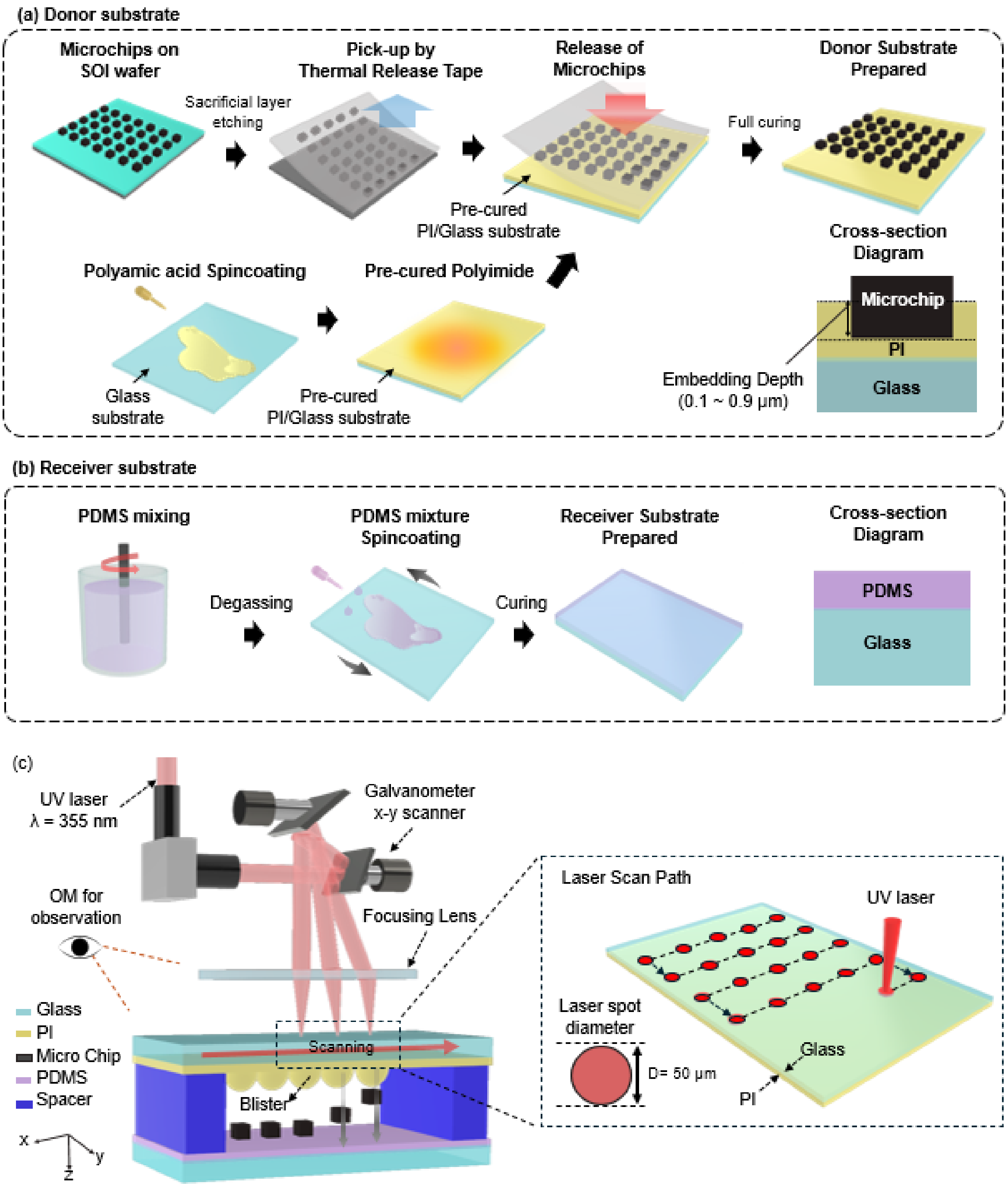

Figure S2. 3D profiler image of a rupture obtained under laser energy density of 1.4 W/cm²

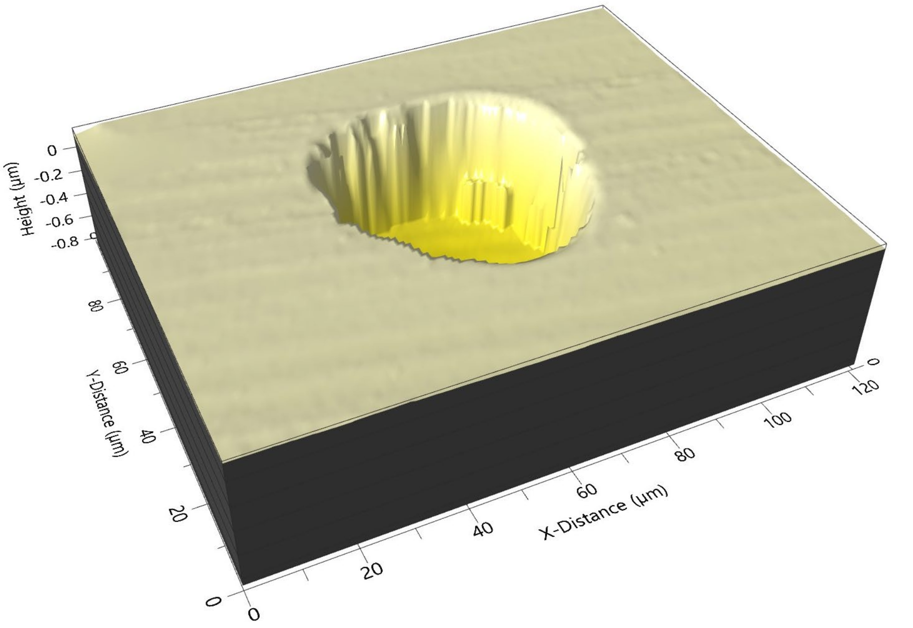

Figure S3. A custom-built optical alignment stage designed to facilitate precise alignment control during the LIFT process. The stage provides adjustments along the x, y, and z axes, as well as rotational control, allowing for accurate alignment of the donor substrate (upper section-Part A) and the receiver substrate (lower section-Part B).

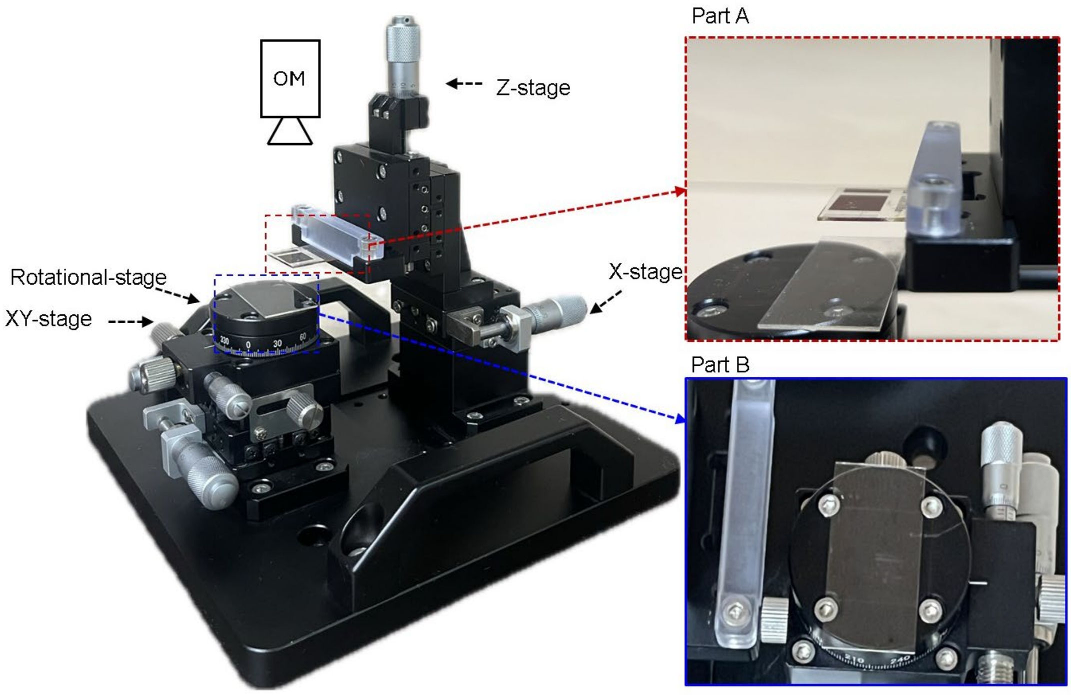

Figure S4. (a) Optical microscopy (OM) image of the donor substrate prior to transfer, highlighting the initial arrangement of microchips. (b) OM image of the substrate after the transfer process, showcasing the transferred microchips. (c) Classification and analysis of transfer outcomes using Python-based image processing: successfully transferred microchips (outlined in green, 65 in total), misaligned microchips (marked in red, 1), and failed microchips (marked in yellow, 15). A grid was overlaid to facilitate precise comparison between the original and final positions, enabling identification of chips deviating by more than 2 μm from the initial spacing of 8 μm or rotating beyond 5°. (d) Measurement of misalignment angles for rotated chips. The black scale bar represents 50 μm.

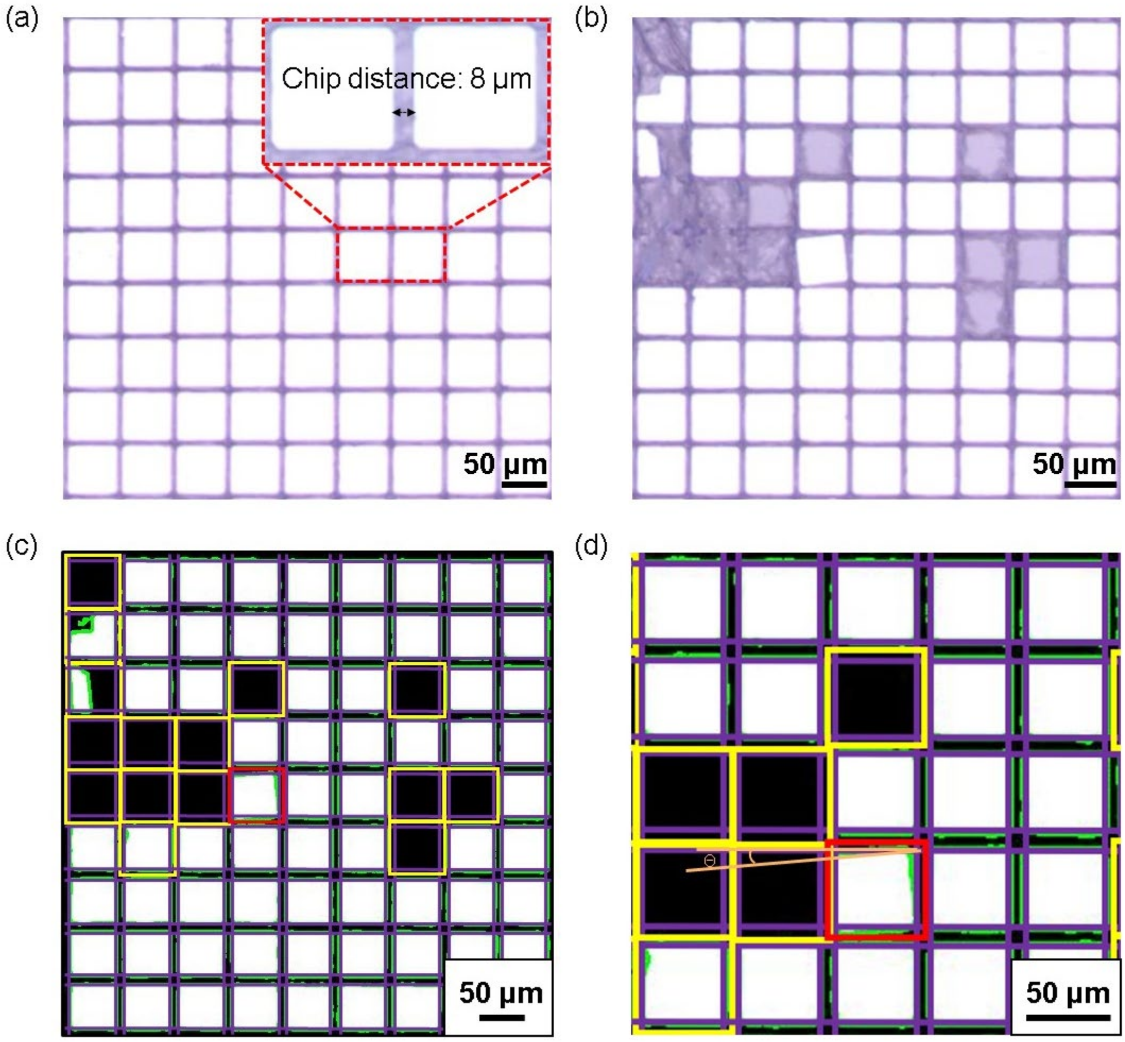

Figure S5. (a) Correlation between the chip embedding depth-to-DRL thickness ratio and transfer yield at a laser energy density of 1.2 Wcm^{-2}. (b) Relationship between dynamic release layer (DRL) thickness and microchip transfer yield under varying laser energy densities (0.6 - 1.4 W cm^{-2}). The graph illustrates the presence of an optimal DRL thickness for each energy density.

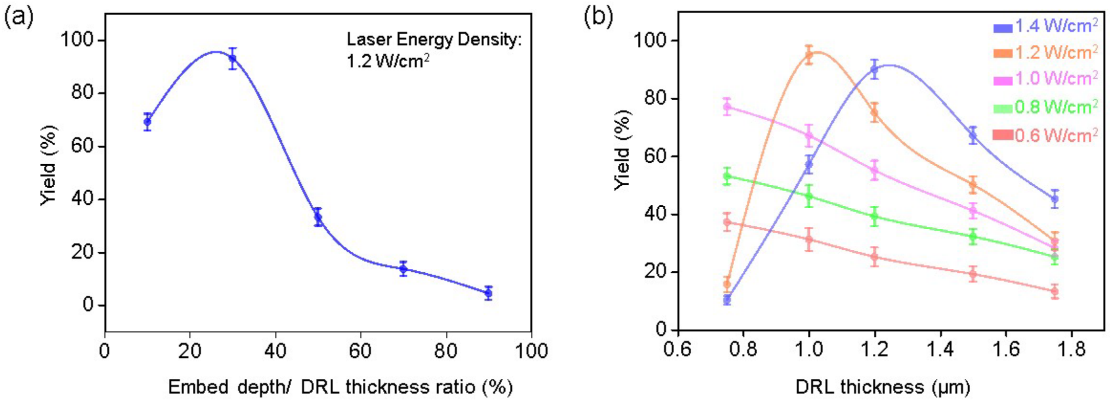

Table S1. Comparison of parameters for blister formation.

Laser Energy Density [W /cm^2]	Diameter/Height [µm]	DRL thickness [µm]	Diameter/Height [µm]	Pulse Width [µm]	Diameter/Height [µm]
**Fixed variable DRL thickness (1 µm) Pulse Width (1.3 µm)**		**Fixed variable Laser Energy Density (1 W/ cm^2) Pulse Width (1 µm)**		**Fixed variable DRL thickness (1.5 µm) Laser Energy Density (1 W/ cm^2)**	
0.6	16 / 0.2 [Blister]	1.0	29.8 / - [Rupture]	1	39.8/ 1.1 [Blister]
0.8	17 / 0.36 [Blister]	1.5	33.8/ 0.88 [Blister]	1.7	45 / - [Rupture]
1.0	21 / 0.47 [Blister]	2.0	51.6 / - [Rupture]	2.4	47 / - [Rupture]
1.2	24 / 0.67 [Blister]	2.5	50.6 / - [Rupture]	3.1	51 / - [Rupture]
1.4	27 / - [Rupture]	13.0	47.6 / - [Rupture]	3.8	55 / - [Rupture]

Table S2. Comparison of parameters for transfer yield.

DRL thickness [µm]	Transfer yield [%]	Embed Depth [µm]	Transfer yield [%]
**Fixed variable Laser Energy Density (1.2 W/ cm^2) Pulse Width (1.3 µm)**		**Fixed variable Laser Energy Density (1.2 W/ cm^2) Pulse Width (1.3 µm) DRL thickness (1 µm)**	
0.75	**15.3** ± 3.13	0.1	**69** ± 3.03
1	**94** ± 3.4	0.3	**93** ± 3
1.25	**66.2** ± 3.31	0.5	**33** ± 3.28
1.5	**56** ± 2.65	0.7	**13.4** ± 2.05
1.75	**30.4** ± 2.41	0.9	**4.2** ± 1.72

Table S3. Comparative analysis of the effect of DRL curing time on the embedding depth of microchips.

**DRL curing Time [min]**	**Embedding depth [µm]**
5	0.9
10	0.7
20	0.5
40	0.3
80	0.1
